# Correction of Peri-Implant Soft Tissue Fenestration With Bony Dehiscence Associated With Intrabony Defect: A 2-Year Case Report

**DOI:** 10.1155/2024/5895661

**Published:** 2024-08-13

**Authors:** Sujiwan Seubbuk Sangkhamanee, Thitiwan Teparat-Burana

**Affiliations:** Department of Oral Medicine and Periodontology, Faculty of Dentistry, Mahidol University, Bangkok 10400, Thailand

**Keywords:** bony dehiscence, connective tissue graft, peri-implant soft tissue fenestration, peri-implantitis

## Abstract

Soft and hard tissue deficiencies around dental implants which can potentially compromise implant survival are commonly encountered. Complicated interventions are often required to address and resolve combinations of soft and hard tissue defects. This case report describes the management of peri-implant soft tissue fenestration accompanied by bony dehiscence associated with intrabony defect through soft tissue modification. A 51-year-old female was referred to the Periodontics and Oral Medicine Clinic with labial soft tissue fenestration at the maxillary left canine implant-supported crown. The patient complained of discomfort and malodor at the implant site. The implant showed mucosal fenestration and 6 mm probing depth (PD) with profuse bleeding at the distolabial site without mobility. A cone beam computed tomography (CBCT) demonstrated labial bony dehiscence associated with a 5.56-mm intrabony defect at mesial and distal surfaces. The implant was diagnosed as peri-implantitis with soft tissue deficiency. The treatment comprised oral hygiene instruction, debridement of the implant and all natural teeth, and mucogingival surgery with free connective tissue graft by the envelope technique. Two weeks after debridement, the mucosal margin of the implant disappeared, presenting soft tissue dehiscence of 4 × 4 mm. Mucogingival surgery was performed 3 weeks later. A 2-year follow-up revealed a stable mucosal margin with PD ranged 2–4 mm. In conclusion, modification of the soft tissue thickness around the implant together with excellent plaque control by the patient successfully maintained peri-implant health.

## 1. Introduction

Nowadays, rehabilitation of edentulism with dental implants provides long-term function and improved esthetics. Soft and hard tissue deficiencies around the implant placement site are common problems caused by several factors including pre-existing tissue morphology, postextraction bone changes, and the condition of the extracted tooth [1]. Tissue deficiencies may impact the efficacy of peri-implant health maintenance and implant stability [2]. Many clinical studies have addressed the prevention of tissue deficiencies as an early step before tooth extraction and the management of defects after implant therapy. Evidence-based clinical recommendation for the management of an extraction socket with alveolar ridge preservation and bone regeneration at the time of implant placement was deliberated, together with the protocol for successful implant therapy [3]. Oral hygiene care, mechanical debridement, and local antiseptic are suggested to prevent peri-implant inflammation [4]. The efficacy of soft tissue augmentation and bone regeneration was systematically reviewed as treatment modalities to correct both soft and hard tissue deficiencies and maintain peri-implant health [5, 6]. Management difficulties occur, especially in the esthetic zone, while implant survival and the esthetic outcome are also points of concern, especially in the anterior maxilla. In this case report, a female patient was diagnosed with mucosal fenestration at the maxillary left canine implant. Bony dehiscence with mesial and distal intrabony defect presented beneath the soft tissue radiographically. The aim of this case report was to demonstrate the management of peri-implant soft tissue fenestration with bony dehiscence associated with intrabony defect.

## 2. Patient Information

A 51-year-old female was referred to the Periodontics and Oral Medicine Clinic, Faculty of Dentistry, Mahidol University, with mucosal fenestration at the labial site of the maxillary left canine implant-supported crown. The patient reported discomfort and malodor at this site. Tissue level implant (Straumann® Standard Plus Implant, diameter 4.1 mm Regular Neck, SLA® 10 mm) had been simultaneously placed 6 years ago with ridge augmentation using Straumann® BoneCeramic 90% porous, 400–700 *μ*m, and a Bio-Gide 13 mm × 25 mm, with a permanent crown fixed using polycarboxylated cement 8 months after implant placement. The patient had rheumatoid arthritis and was taking 12.5 mg of methotrexate a week, 500 mg of sulfasalazine twice daily, 250 mg of naproxen twice daily, 20 mg of omeprazole twice daily, 400 mg of gabapentin three times a day, and 5 mg of folic acid once a day. The patient regularly visited the rheumatologist and had a history of allergy to amoxicillin.

### 2.1. Intraoral Examination

Mucosal fenestration of 3 × 3 mm was noted at the labial implant site, 2 mm apical to the mucosal margin (Figure 1(a)). The implant presented 6 mm PD at the distolabial site with profuse bleeding, while other sites showed 3 mm PD with bleeding on probing (BOP) and no mobility. There was no pus exudation at both implant and natural teeth. All the natural teeth presented 2–4 mm PD with some calculus deposition.

### 2.2. Radiographic Examination

CBCT scan images revealed dehiscence of the labial bone to the apex of the implant and vertical bone loss at mesial and distal surfaces of 5.56 mm (Figure 1(b)).

### 2.3. Diagnosis and Prognosis

The implant was diagnosed as peri-implantitis [7]. The soft tissue deficiency at the implant site was classified as Class 3 according to Parma-Benfenati, Tinti, and Roncati [8] and presented gingival recession and bony dehiscence associated with mesial and distal intrabony defect. The morphology and severity of peri-implantitis bone defect were classified as Class Ib Grade A according to Monje et al. [9]. The pretreatment prognosis was categorized as unfavorable [10] due to the combination of bone loss at more than half of the implant length, BOP, and no mobility.

### 2.4. Treatment

The patient was informed about the prognosis and implant treatment plan at the first visit. The treatment began with oral hygiene instruction using the modified Bass brushing technique and dental flossing. A single tuft brush was introduced for cleaning at the implant site. The patient received full mouth scaling together with implant debridement and polishing. Chlorhexidine (0.12%) mouthwash was prescribed for gargling twice a day. Two weeks after the initial debridement, the patient reported no discomfort and malodor. The labial soft tissue of the implant disappeared and presented as 4 × 4 mm soft tissue dehiscence (Figure 1(c)). The attached gingiva presented 1 mm at the midlabial site, with excellent plaque control. The surgical treatment plan for the implant comprises mucogingival reconstruction by connective tissue graft to increase mucosal thickness and keratinized tissue width, followed by bony reconstruction via guided bone regeneration (GBR). One week later, the connective tissue graft was performed.

### 2.5. Surgical Therapy

The implant area was locally anesthetized with 4% articaine with epinephrine 1:100,000 1.5 cartridges (2.6 mL) by infiltration. The implant surface was debrided and irrigated with 0.12% chlorhexidine mouthwash. The recipient site was prepared by creating a partial thickness envelope flap at the labial aspect of the implant and extended 6–7 mm mesially, distally, and apically (Figure 2(a)). The epithelialized soft tissue graft (size 16 × 8 mm) was harvested from palatal tissue of the upper left first premolar to upper left first molar. The graft was de-epithelialized, except for the area that lay over the exposed implant fixture (Figure 2(b)). The graft was then placed in the previously created envelope to completely cover the implant fixture and secured mesially and distally. The prescribed medication included clindamycin 300 mg, 1 capsule orally every 6 h, and 0.12% chlorhexidine mouthwash with gargling twice a day. Clindamycin was prescribed to prevent postoperative infection because the patient suffered from rheumatoid arthritis, had a history of allergy to amoxicillin, and was taking methotrexate as an immunosuppressant.

### 2.6. Follow-Up

At the 1-week follow-up visit, the patient reported discomfort for a few days after the surgical procedure. The soft tissue around the graft was slightly inflamed and edematous (Figure 3). The donor site presented a small hematoma and partial epithelialization. The sutures were removed, and the patient was instructed to gently wipe the implant soft tissue interface with 0.12% chlorhexidine-soaked cotton pellets.

One week later, the graft was blended with the surrounding tissue. Mucosal gingiva was observed slightly apical to the crown margin, with metal implant neck exposure of less than 1 mm. The patient resumed routine oral hygiene care together with regular chlorhexidine wiping at the implant site.

The patient missed the next follow-up visit due to the COVID-19 pandemic but returned to the clinic 8 months after the surgery. The implant PD showed 3 mm distolabially and midlabially and 5 mm mesiolabially, without BOP or suppuration. The mucosal margin covered the implant neck which indicated some creeping after the connective tissue grafting (Figure 3). The adjacent teeth, upper left lateral incisor, and first premolar presented some chlorhexidine staining because the patient had wiped the implant area with chlorhexidine continuously beginning 1 week after the surgery. Supportive periodontal therapy was performed. The patient presented excellent plaque control and was advised to stop using chlorhexidine at the implant site. At this point, GBR procedure was postponed since the PD around the implant can be maintained within 5 mm without BOP.

At the 13-month and 2-year follow-ups, the implant PD ranged from 2 mm distolabially and midlabially to 4 mm mesiolabially, absence of BOP, and a 4 mm band of keratinized mucosa. The soft tissue margin was stable. CBCT radiographs demonstrated the improvement of the intrabony defect in depth (3.75 mm at mesial and 4.95 mm at distal) and width when compared to the pretreatment (Figure 3). Therefore, the patient was appointed for the 6-month recall program without further GBR.

## 3. Discussion

Alveolar ridge dimensional changes normally occur after tooth extraction. Data from a systematic review [11] showed an alveolar ridge reduction of 3.87 mm in width, 1.67 mm in midbuccal height, and 1.53 mm in radiographic crestal height. The prevalence of buccal peri-implant soft tissue dehiscence was 16.9% [12]. However, prevalence in the esthetic area increased to 54.2% and 56.8% on a patient and implant level, respectively [13]. One of the determining factors of soft tissue contour was the presence of bone [14]. Buccal bone thickness should be at least 2 mm. If this thickness of bone is not available, some part of the buccal plate will be lost after remodeling, leading to a high risk of soft tissue recession. Risk indicators of peri-implant soft tissue dehiscence in the esthetic area, besides the presence of an adjacent implant, included increased time in implant function and higher buccal bone distance, leading to lower keratinized mucosa width and mucosal thickness [13], while the thickness of soft tissue significantly reflected marginal bone loss. Implants with thin soft tissue presented higher marginal bone loss than implants with thick soft tissue [1]. A long-term study over 10 years of early implant placement with simultaneous contour augmentation in anterior single-tooth sites also found a significant correlation between facial bone crest height and soft tissue thickness [15]. Median peri-implant bone loss was 0.35 mm with a success rate of 95%, with the 5% failure rate presenting no facial bone wall and vertical bone loss of 5.92 mm at the 6-year and 10-year follow-ups. In this case report, the patient had simultaneous implant placement in combination with GBR at the upper left canine. The implant presented both soft and hard tissue dehiscence within 7 years after prosthetic loading. Peri-implant tissue including a thin biotype and narrow band of keratinized mucosa may have affected soft and hard tissue deficiency in this case. A higher incidence of facial fenestration and dehiscence defects was also reported in canine teeth, with greater angulation between the long axis of the tooth and the midline of the ridge than in the other anterior teeth [16].

The treatment of peri-implantitis can include both nonsurgical and surgical techniques. A literature review by Renvert, Roos-Jansaker, and Claffey suggested that mechanical nonsurgical therapy was effective in peri-implant mucositis treatment, while similar effectiveness was not found in peri-implantitis [17]. However, a 7-year longitudinal clinical case report found remarkable changes in bacterial composition after nonsurgical treatment of peri-implantitis with horizontal bone loss [18]. The 5-year follow-up showed a decrease in *Porphyromonas*, *Prevotella*, *Alloprevotella*, and *Tannerella* genera in phylum Bacteroidetes and the genus *Fusobacterium* in phylum Fusobacteria. By contrast, the genus *Streptococcus* in phylum Firmicutes significantly increased. These results showed the effect of nonsurgical treatment in promoting microbiota recovery from dysbiosis. The use of adjunctive local or systemic antibiotics also reduced BOP and PD [17]. To resolve peri-implant recession or soft tissue fenestration with severe buccal dehiscence or interproximal bone loss, a two-stage technique was recommended [8]. Initial mucogingival surgery should be followed by osseous reconstruction 4–6 months later. A retrospective clinical cohort study [19] showed increased keratinized tissue width from 0.4 ± 0.5 mm to 4.3 ± 1.5 mm and reduced PD from 6.3 ± 2.3 to 4.1 ± 1.9 mm after soft tissue grafting. Our result presented increased width of keratinized tissue to 4 mm, as found in the previous study, while PD reduced from 6 to 2 mm, higher than the previous report [19]. This case report suggested no further regenerative osseous surgery since the PD was maintained at 2–4 mm throughout 2 years, with excellent oral hygiene care by the patient. The mucogingival approach allows self-filling to compensate for the neighboring bone defect [8].

Several techniques are available for plastic surgical reconstruction of peri-implant tissue. The soft tissue graft can be either an autogenous graft or a soft tissue substitute. Autogenous grafts were favored to maintain peri-implant health by increasing keratinized tissue width and mucosal thickness [5]. Autogenous soft tissue grafts, both free gingival graft and connective tissue graft, were used to augment peri-implant keratinized tissue width and soft tissue thickness. Connective tissue graft was primarily indicated for peri-implant soft tissue thickness augmentation and peri-implant soft tissue dehiscence correction [20]. A prospective cohort study showed 99.2% mean soft tissue dehiscence coverage and 79% complete dehiscence coverage, with significantly increased keratinized tissue width and soft tissue thickness at 5 years after treatment of buccal soft tissue dehiscence around a single implant with a coronally advanced flap in combination with a connective tissue graft [21]. The study also mentioned the evidence of “creeping,” with soft tissue thickness and dehiscence coverage increasing between 1 and 5 years. Furthermore, an envelope flap was suggested instead of a pouch flap to resolve facial defects larger than 2 mm [22]. Therefore, an envelope flap was chosen in our case study since the dehiscence defect was 5.56 mm according to CBCT.

The healing of coronally advanced flap in combination with connective tissue graft around dental implant sites was observed by Doppler ultrasonography [23]. The blood flow at 1 week and 1 month is predominantly from the flap towards the graft. Early tissue perfusion of the graft significantly associated with the higher final mean coverage of peri-implant soft tissue dehiscence and mucosal thickness gain. In this case study, following the mucogingival reconstruction, the implant exhibited full coverage with increased mucosal thickness.

There are some limitations in this case report involving only one patient demonstrating successful treatment of peri-implantitis using connective tissue graft. Success was determined based on clinical appearance. At the 2-year follow-up visit, the implant presented a stable mucosal margin with improved soft tissue thickness, an excess band of keratinized tissue, PD < 5 mm, and no BOP around the implant site. The CBCT radiographs also showed the improvement of the intrabony defect depth and width, although there was the remaining intrabony defect. The presence of a healthy mucosal seal may explain this, as it could resist probe penetration. The patient was satisfied with the therapeutic outcome and compliant with the supportive periodontal therapy, except during the period of the COVID-19 pandemic.

In conclusion, modification of the soft tissue thickness around the implant site was achieved by the combination of a subepithelial connective tissue graft and a coronally advanced flap to correct peri-implant soft tissue fenestration and maintain peri-implant health. One important factor was the patient's excellent oral hygiene care, which led to a successful treatment outcome in this case report.

## Figures and Tables

**Figure 1:**
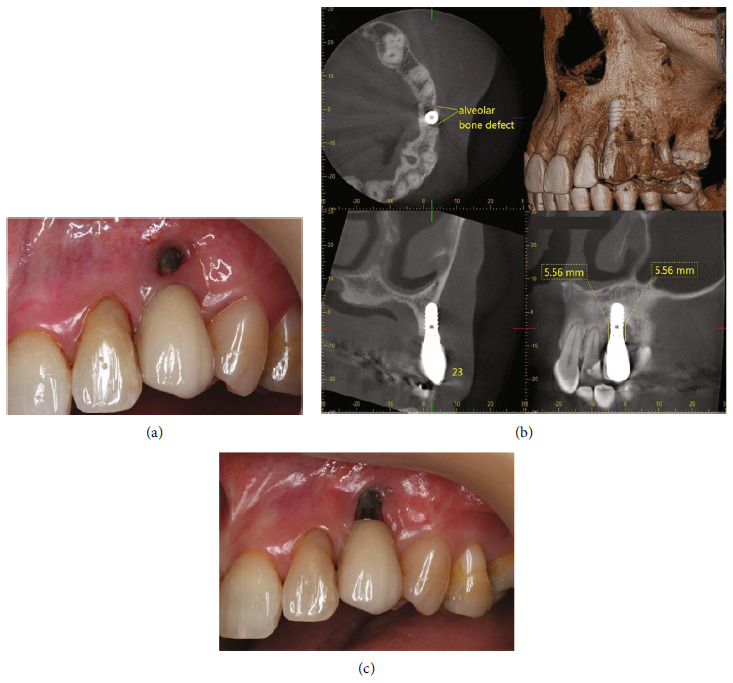
(a) Soft tissue fenestration of 3 × 3 mm at the labial site of the maxillary left canine implant, 2 mm apical to the mucosal margin. (b) CBCT radiographs showing apical labial bony dehiscence and vertical bone loss of 5.56 mm in depth at the mesial and distal surfaces of the implant. (c) The implant presented soft tissue dehiscence of 4 × 4 mm exposing the fixture at 2 weeks after initial debridement.

**Figure 2:**
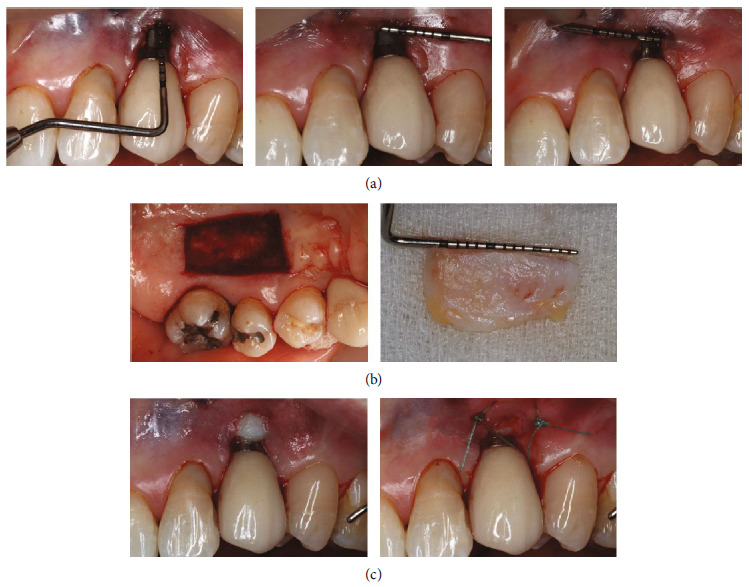
(a) Recipient site with partial thickness flap creating an envelope. (b) Donor site for harvesting the soft tissue graft of 16 × 8 mm. (c) Graft placement.

**Figure 3:**
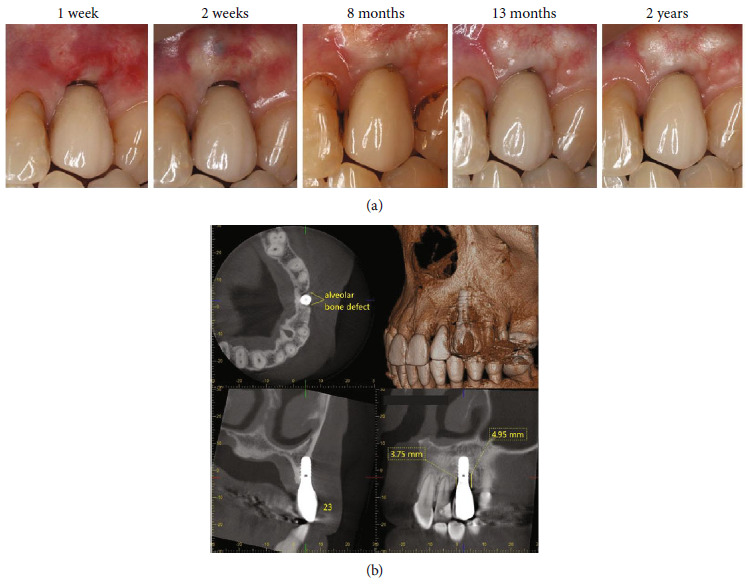
(a) Clinical appearance at the follow-up periods after surgery. (b) CBCT radiographs at 2 years after surgery demonstrating the remaining intrabony defect depth 3.75 mm at the mesial and 4.95 mm at the distal surfaces of the implant. The width of the intrabony defect was narrower than the pretreatment.
